# Results of a Culturally Tailored Smartphone-Delivered Physical Activity Intervention Among Midlife African American Women: Feasibility Trial

**DOI:** 10.2196/27383

**Published:** 2021-04-22

**Authors:** Rodney P Joseph, Barbara E Ainsworth, Kevin Hollingshead, Michael Todd, Colleen Keller

**Affiliations:** 1 Center for Health Promotion and Disease Prevention Edson College of Nursing and Health Innovation Arizona State University Phoenix, AZ United States; 2 College of Health Solutions Arizona State University Phoenix, AZ United States; 3 Department of Kinesiology Shanghai University of Sport Shanghai Shi China; 4 Edson College of Nursing and Health Innovation Arizona State University Phoenix, AZ United States

**Keywords:** exercise, physical activity, minority health, women’s health, mHealth, mobile phone

## Abstract

**Background:**

Regular aerobic physical activity (PA) is an important component of healthy aging. However, only 27%-40% of African American women achieve national PA guidelines. Available data also show a clear decline in PA as African American women transition from young adulthood (ie, 25-44 years) into midlife. This decline in PA during midlife coincides with an increased risk for African American women developing cardiometabolic disease conditions, including obesity, type 2 diabetes, and cardiovascular disease. Thus, effective efforts are needed to promote PA among sedentary African American women during midlife.

**Objective:**

This study aims to examine the acceptability and feasibility of a culturally tailored, smartphone-delivered PA intervention, originally developed to increase PA among African American women aged 24-49 years, among a slightly older sample of midlife African American women aged 50-65 years.

**Methods:**

A single-arm pretest-posttest study design was implemented. In total, 20 insufficiently active African American (ie, ≤60 min per week of PA) women between the ages of 50-65 years participated in the 4-month feasibility trial. The *Smart Walk* intervention was delivered through the study *Smart Walk* smartphone app and text messages. Features available on the *Smart Walk* app include personal profile pages, multimedia PA promotion modules, discussion board forums, and an activity tracking feature that integrates with Fitbit activity monitors. Self-reported PA and social cognitive theory mediators targeted by the intervention (ie, self-regulation, behavioral capability, outcome expectations, self-efficacy, and social support) were assessed at baseline and at 4 months. Feasibility and acceptability were assessed using a postintervention satisfaction survey that included multiple-choice and open-ended questions evaluating participant perceptions of the intervention and suggestions for intervention improvement. Wilcoxon signed-rank tests were used to examine pre- and postintervention changes in the PA and social cognitive theory variables. The effect size estimates were calculated using the Pearson *r* test statistic.

**Results:**

Participants increased moderate-to-vigorous PA (median 30 minutes per week increase; *r*=0.503; *P*=.002) and reported improvements in 2 theoretical mediators (self-regulation: *r*=0.397; *P*=.01; behavioral capability: *r*=0.440; *P*=.006). Nearly all participants (14/15, 93% completing the satisfaction survey) indicated that they would recommend the intervention to a friend. Participants’ suggestions for improving the intervention included enhancing the intervention’s provisions of social support for PA.

**Conclusions:**

The results provide preliminary support for the feasibility of the smartphone-based approach to increase PA among midlife African American women. However, before larger-scale implementation among midlife African American women, enhancements to the social support components of the intervention are warranted.

**Trial Registration:**

ClinicalTrials.gov NCT04073355; https://clinicaltrials.gov/ct2/show/NCT04073355

## Introduction

### Background

African American women are disproportionally burdened by cardiometabolic diseases. Overall, 55% of African American women are obese [1], 57% have cardiovascular disease [2], and 13% have diabetes [3]. These cardiometabolic diseases affect African American women at higher rates than White and Hispanic women (ie, among non-Hispanic White women, 38% are obese, 43% have cardiovascular disease, and 7% have diabetes; prevalence rates among Hispanic women are 51%, 43%, and 12%, respectively). The high rates of these conditions among African American women contribute to their relatively high cardiovascular mortality rate, which is 2 times greater than that of non-Hispanic White and Hispanic women [4].

Recent data from 3 national data sets (ie, Behavioral Risk Factors Surveillance System, National Health and Nutrition Examination Survey, and National Health Interview Survey) indicate that only 27%-40% of African American women meet national aerobic physical activity (PA) guidelines (ie, 150 min per week of moderate-intensity PA, 75 min of vigorous PA, or an equivalent combination of durations and intensities [5,6]). These data also show a decline in PA as African American women transition from young adulthood (ie, 25-44 years) into midlife and older age [6]. The decline of PA among African American women during midlife is particularly concerning, given physiological changes associated with menopause (eg, decreased estrogen and progesterone production) that further increase a woman’s risk of developing cardiometabolic diseases [7-9]. Thus, intervening to promote PA among sedentary African American women during midlife is critical to reducing cardiometabolic health disparities in this population.

Evidence accumulated over the past decade has shown that PA interventions delivered through mobile and smartphone devices (commonly referred to as mobile health [mHealth] PA interventions) are effective for promoting PA [10-12]. However, few mHealth PA interventions have focused on African American women [13-16], and even fewer have included midlife or older African American women [14,15]. This represents a missed public health opportunity, given the low PA levels, high cardiometabolic disease burden, and high level of smartphone use among African American women (recent data show that 79% of adults between the ages of 50 and 65 years own a smartphone, with limited or no differences by sex, race, or ethnicity [17]).

In a previous study, we developed *Smart Walk*, a 4-month culturally tailored, social cognitive theory (SCT)—based smartphone-delivered intervention designed to increase PA and reduce cardiometabolic disease risk among African American women aged 24 to 49 years (ClinicalTrials.gov NCT02823379) [18]. *Smart Walk* was initially developed for women aged 24 to 49 years because it allowed us to tailor the PA intervention to the social, cultural, and behavioral characteristics of young to midlife African American women, who, at the time when the project was conceived (ie, in 2013), were the most likely age group to own a smartphone [19]. However, since *Smart Walk* was funded in 2015, smartphone use among midlife and older adults has increased substantially [17]. Similarly, recruitment efforts for the original *Smart Walk* pilot trial resulted in numerous women aged >50 years contacting our research team and expressing the desire to participate in the research study, indicating a demand for smartphone-delivered health promotion research among African American women aged ≥50 years.

### Objectives

This study aims to examine the feasibility and acceptability of the *Smart Walk* intervention among African American women aged 50-65 years. We hypothesized that data collected from the study would support the acceptability and feasibility of the approach and provide information on how the intervention can be refined to meet the social, cultural, and behavioral norms and preferences of women in this age group.

## Methods

### Study Design and Participants

This study was registered with ClinicalTrials.gov identifier NCT04073355. A single-arm pretest-posttest design was implemented. In total, 20 insufficiently active African American women with obesity (ie, BMI≥30 kg/m^2^) aged between 50 and 65 years were recruited to participate in the 4-month smartphone-delivered PA intervention. Inclusion criteria were as follows: (1) self-identifying as African American and female, (2) aged 50-65 years, (3) having a BMI ≥30 kg/m^2^, (4) performing ≤60 minutes per week of moderate- to vigorous- intensity physical activity (MVPA) according to the 2-item Exercise Vital Sign Questionnaire [20], and (5) no self-reported ambulatory issues associated with moderate-intensity walking. Exclusion criteria included (1) concurrent participation in another PA, nutrition, or weight loss program at the time of enrollment or any time during the 4-month study and (2) indication of a potential contraindication of exercise according to the 2015 PA Readiness Questionnaire [21], unless a physician note allowing participation was provided. All study procedures were approved by the Institutional Review Board of Arizona State University.

### Description of the Smart Walk Intervention

*Smart Walk* is a culturally tailored, SCT—based 4-month PA intervention delivered through the *Smart Walk* smartphone app and text messages. The behavioral PA goal of the intervention was for participants to meet national guidelines of 150 minutes per week of at least moderate-intensity PA, with walking emphasized as the primary behavior to achieve this goal. An overview of the *Smart Walk* intervention is given below. Recent publications by our research team present in-depth descriptions of the development process [22] and design of the intervention [18].

#### Smart Walk Smartphone App

The *Smart Walk* app includes 4 main features designed to promote daily PA: (1) personalized profile pages, (2) multimedia PA promotion modules delivered on a weekly basis in the form of brief videos and electronic text with images, (3) discussion boards for participants to discuss the weekly PA modules and give or receive social support, and (4) a PA self-monitoring or tracking tool that integrates with Fitbit (Fitbit Inc) activity monitors. Screenshots of app features were present in a study by Joseph et al [18].

#### Personal Profile Pages

This feature was designed to be similar to profile pages available on commercial social media websites (eg, Facebook and Twitter) and provided participants with a platform to share select biographical information (ie, picture name, age, neighborhood or local area of residence, and brief biographical narrative) with other study participants. Profiles were designed to help facilitate building of a web-based community and social support network for PA.

#### Multimedia PA Promotion Modules

Weekly multimedia video and text-based modules were the primary delivery channels for the educational and behavioral components of the program. These modules consisted of text- and image-based PA promotion materials written at less than eighth-grade reading level and brief 3- to 7-minute videos, narrated by the study’s African American spokesperson (an African American woman in her mid-30s). Table 1 lists the module topics for the PA promotion program. During the first 3 months of the intervention, new PA promotion modules were delivered weekly. During month 4, new modules were presented every 2 weeks and focused on the maintenance of PA after the active intervention phase.

**Table 1:** Weekly physical activity topics covered during the intervention.

Week number	Module number	Module topic—PA^a^ group
1	1	Introduction to the national PA guidelines and the health benefits of PA
2	2	Overview of PA-related health disparities among African American women and the importance of being a PA role model
3	3	Time management and strategies for incorporating 30 min of PA into the day
4	4	PA goal setting
5	5	Overcoming general barriers to PA
6	6	Tips for increasing daily PA
7	7	Overcoming hair care barriers to PA
8	8	Creating a social support network for PA
9	9	Trying new types of activities
10	10	Reducing sedentary time
11	11	Dietary behaviors to complement PA
12	12	Muscle strengthening and stretching activities to complement aerobic PA
13	N/A^b^	N/A
14	13	Dealing with setbacks
15	N/A	N/A
16	14	Review of previous modules and maintenance of PA after the active intervention phase

^a^PA: physical activity.

^b^N/A: not applicable.

#### Discussion or Message Boards

Weekly PA promotion modules were accompanied by relevant discussion forum topics to encourage participants to reflect on the information presented in the multimedia PA promotion modules, share their personal experiences about PA, and give and receive social support for PA. The discussion board feature also included a general *Community Board* forum and a *Meet-up* forum, where participants could share information and/or discuss topics that may not clearly align with the weekly module topics and discuss or coordinate group-based exercise activities. The dialog on these discussions was a primary mechanism through which social support for PA was fostered among participants. Push notifications were sent to study participants when other participants posted on the discussion boards. Example module discussion board topics are as follows: “Why do you want to be physically active?” and “What are ways you add more physical activity into your day?”

#### PA Self-Monitoring or Goal Setting Feature

Participants received a wrist-worn Fitbit Inspire HR (Fitbit Inc) activity monitor to wear throughout the study. Data collected from the Fitbit integrates with the *Smart Walk* app to allow participants to view and track the minutes of MVPA performed during the study. The criterion used to define MVPA, as measured by the Fitbit, was walking at a cadence of 100 steps per minute [23,24] for at least 1 minute. Activity not meeting this intensity-duration criterion was not registered as MVPA on the *Smart Walk* app. For activities not recorded by the activity monitor (eg, stationary cycling, water aerobics, and swimming), participants could manually enter the activity via the app’s tracking feature. Fitbits were registered to a research study account to allow for staff to monitor PA progress and troubleshoot any device-related issues experienced by participants.

#### PA Promotion Text Messages

Participants received 3 PA promotional text messages each week. These text messages provided participants with inspirational quotes, reminders, and tips for increasing daily PA. Message content was developed through our formative research with African American women [25,26]. Example text messages are as follows: “Commit to being fit. It’s never too late to achieve your physical activity goals.” and “Be an active role model to those around you!”

#### Theoretical Basis of the Intervention

SCT [27] served as the theoretical framework for the intervention. Developed by Bandura [27], SCT posits that human behavior is a result of the reciprocal and dynamic interaction of personal factors and the social environment. Intervention components were designed to engage 5 constructs of SCT: behavioral capability, social support, self-efficacy, outcome expectations, and self-regulation. Textbox 1 provides a brief description of each of these constructs and how the intervention was designed to leverage each construct to promote PA.

**Social cognitive theory constructs targeted by the intervention.**

Behavioral capability—knowledge and skill to perform a physical activity (PA)

- Multimedia modules provide information on:
  Definitions of PA and exercise
  Different types of PA (ie, aerobic vs muscle strengthening)
  National PA guidelines and the types of PA that can be performed to achieve the PA guideline
  How to determine the intensity of PA performed

Social support—extent to which significant referents approve, encourage, and/or influence performance of PA

- Discussion board prompts facilitate and encourage participants to give and receive emotional support for PA
- Multimedia modules and text messages:
  Provide participants with encouragement and empowerment for PA
  Emphasize PA is a form of self-care and that African American women are worthy of self-care activities
  Encourage participants to reflect on why they should be physically active
  Include talking points and negotiation strategies to facilitate social support for PA

Self-efficacy—confidence in oneself to take action and overcome barriers. Self-efficacy is derived through 4 main sources: (1) Mastery experiences (ie, first-hand experience with performing a behavior), (2) Social modeling (ie, seeing other similar to ourselves successfully perform a behavior), (3) Emotional arousal (ie, improving emotional states by reducing stress or anxiety and by promoting positive emotions), and (4) Verbal persuasion (ie, verbal encouragement to engage in a behavior):

- Participants track PA using the activity tracking feature, allowing participants to enact PA strategies encouraged by the intervention and track their increases or decreases in PA (ie, mastery experiences)
- Multimedia modules:
  Illustrate African American women engaging in various types of aerobic PA and include testimonials from African American women describing how and why they are physically active (ie, social modeling)
  Reinforce the idea that PA does not have to be structured or difficult by encouraging walking and providing tips on how more walking can be incorporated into day (ie, emotional arousal)
- Multimedia modules and text messages encourage participants to be active by providing words of encouragement and empowerment for PA (ie, verbal persuasion)
- Discussion boards and weekly prompts provide a venue for participants encourage each other to be physically active (ie, verbal persuasion)

Outcome expectations—anticipated outcomes of engaging in PA

- Weekly video and text modules provide participants with the health and social outcomes associated with being physically activity, including:
  Reduced risk for heart disease and type 2 diabetes
  Weight maintenance
  More energy to perform daily activities
  Improved quality and quantity (ie, years) of life
  Being a good role model to others

Self-regulation—ability to manage social, cognitive, and motivational processes to achieve a desired goal

- Participants use Fitbit activity monitor and *Smart Walk* PA self-monitoring feature to track daily and weekly PA.
- Intervention materials encouraged to achieve the static goal of 150 min per week of moderate- to vigorous-intensity PA. In addition, the week 4 module encourages participants to create short- and long-term goals associated with PA performance.
- The week 4 module encourages participants to create self-rewards for achieving previously established PA goals.

#### Cultural Tailoring of the Intervention

Cultural tailoring of the intervention was informed through an extensive review of the literature and our formative focus group research with adult African American women (mean age of focus group participants was 38.5 years, SD 7.8). The intervention was designed to be sensitive to, and to leverage, the lived experiences, sociocultural norms, and familial and societal expectations of African American women for the successful promotion of PA. On the basis of the cultural tailoring framework by Resnicow et al [28], the intervention addressed both *surface* and *deep-structure* cultural characteristics of African American women. Surface-level cultural tailoring refers to the most basic level of cultural tailoring and includes matching the characteristics and packaging of a promotion to the overt social and behavioral characteristics of the intended population [28]. Surface-level cultural tailoring of the *Smart Walk* was achieved through (1) evidential statistics emphasizing the low levels of PA and high prevalence of cardiometabolic disease conditions among African American women (eg, “Only 36% of African American women meet the National PA Guidelines” and “Almost half (49%) of African American women over the age of 20 have heart disease”), (2) images of African American women with diverse physical characteristics (body shapes, hairstyles, and skin tones) performing PA throughout PA module text, and (3) having a local African American woman serve as the study spokesperson in the video modules. Deep-structure cultural tailoring involves recognizing sociocultural norms and values, beliefs, and behaviors of a group and using these characteristics to motivate behavior change [28]. Deep-structure cultural tailoring efforts focused on 3 key concepts: (1) collectivism or ethics of care, (2) physical appearance norms (ie, hair care or body shape concerns), and (3) racial pride or role modeling. Table 2 describes each cultural consideration and how the intervention was designed to influence these characteristics to promote PA.

**Table 2:** Deep-structure cultural tailoring characteristics of the intervention.

Cultural consideration	Brief description	How the cultural characteristic was addressed in the intervention
Collectivism	Prioritizing the needs and well-being of others (ie, family or friends) over the needs and well-being of oneself. This can contribute to African American women reporting lack of time, energy, or resources for PA^a^. Although this perspective has been reported among women of other races or ethnicities, previous research, including our own pilot work, suggests this phenomenon may be more accentuated in the African American community.	Intervention materials: recognized the importance of caretaking in the value system of African American women. emphasized PA is an investment in the health and well-being of African American women and not a competing interest with caretaking, familial, or other responsibilities. portrayed regular PA as a key behavior to help participants perform their caretaking, familial, and community responsibilities with more energy and for a longer duration throughout the life span.
Racial pride or role modeling	Awareness and interest in how one’s behavior can contribute the collective health and well-being of the African American community.	Intervention materials highlighted that physically active African American women are positive role models to other members of the African American community, which can encourage others in their community (ie, family and friends) to adopt a physically active lifestyle.
Physical appearance preferences	Some African American women are hesitant to engage in PA because perspiration or sweat negatively impacts their hairstyle. they perceive PA will alter their desired body shape.	Intervention materials: included hairstyling strategies to help reduce the negative effects of perspiration (ie, use of hair wraps and dry shampoo strategies). encouraged women to adopt hairstyles that are less impacted by perspiration (ie, braids and natural hairstyles). informed participants that engaging in PA at the levels recommended by the study (ie, 150 min per week) will not substantially change their body shape unless they also change their dietary habits. emphasized health benefits of PA independent of weight loss (ie, reduced cardiometabolic disease risk, weight maintenance, and increased energy).

^a^PA: physical activity.

### Measures

#### Demographic Characteristics

Demographic characteristics (age, education, income, ethnicity, number of children in household, and marital or relationship status) were assessed using a self-administered questionnaire.

#### Anthropometrics

Weight, height, and waist circumference were measured by a member of the research team in a private room while wearing light clothing but no shoes. Body weight was measured in kilograms using the Tanita TBF-300A (Tanita Corporation) digital scale. Height was measured in centimeters using a Seca 213 portable stadiometer. BMI was calculated as weight in kilograms divided by height squared (in meters).

#### PA Outcomes

Self-reported weekly minutes of MVPA was assessed using the 2-item Exercise Vital Sign Questionnaire [20], and weekly energy expenditure was assessed using the REGICOR questionnaire [29]. The Exercise Vital Sign Questionnaire assesses the frequency (days per week) and duration (min per day) of MVPA performed (eg, a brisk walk) during the past week. The questionnaire is scored by multiplying the days by minutes per day of PA to generate an estimate of minutes per week of MVPA. This measure has been validated against population-based surveillance surveys [20] and accelerometers for the accurate assessment of PA [30]. The REGICOR questionnaire is a 16-item questionnaire that assesses leisure-time PA (including time spent in active transportation to work, walking, climbing stairs, and playing sports), time sedentary behaviors, and a ranking of occupational PA. This provides an estimation of total energy expenditure as well as energy expended in light-intensity (<4 metabolic equivalent [MET]), moderate-intensity (4-5.5 MET), and vigorous-intensity (>5.5 MET) activities. Energy expenditure for leisure-time PA is expressed as MET-minute obtained by multiplying the MET intensity level by minutes reported for the activities. The scoring algorithm was described by Molina et al [29]. Here, we only report the results for leisure-time PA, as the study was not designed to target sedentary behavior or occupational PA. The REGICOR has high test-retest reliability for assessing MVPA (interclass correlation=0.79 and 0.95 for moderate- and vigorous-intensity PA, respectively), is sensitive to assessing changes in PA over time, and has been validated against accelerometers [29].

#### SCT Outcomes

SCT constructs were assessed using a series of questionnaires developed and tested in previous research. Self-efficacy for PA was assessed using the 12-item Exercise Confidence Survey [31]. Social support for exercise was evaluated using the 20-item Social Support for Exercise Survey [32]. This scale consists of separate 10-item measures of family support and friend support. Self-regulation for PA was measured using the 10-item Self-Regulation Scale from the Health Beliefs Survey [33,34]. Outcome expectations for PA were assessed using the 9-item Outcome Expectation Scale for Exercise [35]. Behavioral capability for PA was measured using a 5-item scale developed by our research team, adapted from previous research [36,37] to assess knowledge of national PA guidelines and the health benefits of PA.

#### Acceptability and Feasibility

Acceptability and feasibility were assessed by examining objectively measured indices of Fitbit wear, app use, and text message delivery and by evaluating self-reported treatment acceptance via a consumer satisfaction survey. Fitbit wear was assessed by examining the daily Fitbit wear. The criterion used to indicate daily wear was modeled after work by Hartman et al [38] and required participants to accumulate at least 1 minute of light- or moderate-intensity PA (as classified via Fitbit’s tracking algorithm) on a given day. This criterion was selected because participants were instructed to wear the activity monitor to track their activity, as opposed to wear the device all day. Accordingly, achieving 1 minute of activity would indicate that the activity monitor was worn for at least part of the day. App use was assessed using analytic tracking software built into the app during development. Specific data points (events) recorded by this software included (1) when a participant loaded a PA module on her phone, (2) when a participant played a module, and (3) when a participant posted in a discussion board forum. Weekly text messages were sent to participants using Twilio, a commercial cloud-based communication platform, which integrated with the smartphone app platform. The app platform recorded the time and date when a text message was delivered to a participant. In the event that a text message was not delivered, an error note was sent to the research team. Treatment acceptance was assessed using a 37-item consumer satisfaction survey adapted from previous research [13,39]. This survey included both multiple-choice and open-ended questions evaluating participants’ perceptions of the intervention, including content and app usability, and suggestions for how the research team could improve the intervention. Example questions include “Overall, how helpful did you find the weekly videos and text modules to promote physical activity?” and “If we were to do the program again, what do you recommend we change, add, or remove from the program?”

### Procedures

Community-based recruitment strategies were used to recruit participants, including social media posts, newspaper advertisements, in-person recruitment at community events, and word-of-mouth. Women interested in study participation contacted the research team via email, telephone, or the study website (which was included in recruitment materials) and completed an eligibility screening survey by telephone or via the internet. Eligible women were scheduled to attend an in-person study orientation session held on the downtown campus of Arizona State University. This orientation session was designed to provide detailed information about the study activities and allow participants to have any questions regarding the study answered by the study staff. At the conclusion of the orientation session, women who indicated a desire to participate provided written informed consent; completed paper-based baseline surveys; and had their resting blood pressure, height, and weight assessed by study staff. To reduce participant wait time between baseline assessments and initiation of the intervention, participants were enrolled in 3 separate cohorts: cohort 1 included 9 participants who received the intervention from October 2019 to February 2020, cohort 2 included 5 participants enrolled from November 2019 to March 2020, and cohort 3 included 6 participants who received the intervention from December 2019 to April 2020. Before the start of each cohort, a member of the study team provided participants with a Fitbit activity monitor to wear on their nondominant wrist during the intervention and assisted each participant with downloading the *Smart Walk* app. Study staff proactively followed up participants every 2 weeks during the intervention to identify any issues participants may have with the Smart Walk app or Fitbit activity monitor. This contact also served as an opportunity to remind participants to wear the Fitbit daily.

Follow-up study assessment procedures were designed to be similar to baseline. Specifically, participants were expected to attend in-person assessments to complete the study surveys and have their weight and resting blood pressure assessed. However, in-person assessments were not possible for cohorts 2 and 3 because of safety concerns associated with the transmission of SARS-CoV-2 (COVID-19). Accordingly, participants in these 2 cohorts were mailed the 4-month survey packet. After completing the surveys, participants were asked to return the study materials to the research team using a prepaid mailer. However, several participants reported that they were unable to mail the study packet to the research team because they either lived in residences that did not have a mailbox large enough to place the prepaid mailing envelope and/or were unable to access a postal service dropbox. For these individuals, a member of the research team coordinated a no-contact pick-up of the survey and accelerometer at the participant’s residence. Due to the remote nature of follow-up data collection procedures for cohorts 2 and 3, blood pressure and anthropometric data were not collected for these participants. Accordingly, these data are not presented at follow-up because of the large amount of missing data at the 4-month assessment. Participants were provided US $50 for study participation (US $25 after the baseline assessment and US $25 after the 4-month assessment) and were allowed to keep the Fitbit provided to them during the intervention.

### Sample Size Considerations

This study focused on examining the feasibility of the smartphone-delivered intervention among a sample of midlife African American women, rather than efficacy testing. We selected a sample size of 20 because it would yield sufficient information regarding the feasibility of implementing the intervention with a sample of midlife African American women and allow for a preliminary examination of pre- versus postintervention differences in outcomes.

### Statistical Analysis

Descriptive statistics (mean, median, percentage, and frequency) were used to summarize demographic characteristics and postintervention consumer satisfaction survey responses. Due to the small sample size and concerns about data not meeting distributional assumptions for parametric statistical tests, nonparametric techniques were used to examine baseline to 4-month changes in SCT and PA outcomes. With baseline values carried forward for missing data at follow-up, Wilcoxon signed-rank tests were used to examine pre- versus postintervention changes in study outcomes, and correlation coefficients were used to characterize the magnitude of change. Statistical significance was set at a *P* value of <.05; however, *P* values are provided for reference only, as the study was not powered to detect significant changes in study outcomes. Qualitative data provided by participants on the consumer satisfaction survey were analyzed using direct content analysis [40]. For this analysis, participants’ responses to open-ended survey questions were initially coded based on the specific intervention component of the qualitative narrative referenced (multimedia modules, discussion boards, Fitbit, activity tracker, and text messages). Narratives not focused on a specific intervention component were coded into a category broad category entitled *overall satisfaction with the intervention*.

Next, narratives within each code were reviewed, and repetitive themes emerging from the coded data were used to reflect participant sentiments regarding the overall intervention as well as specific intervention components. A qualitative analysis was performed by one member of the research team (RPJ).

## Results

### Participant Flow and Baseline Characteristics

**Figure 1:**
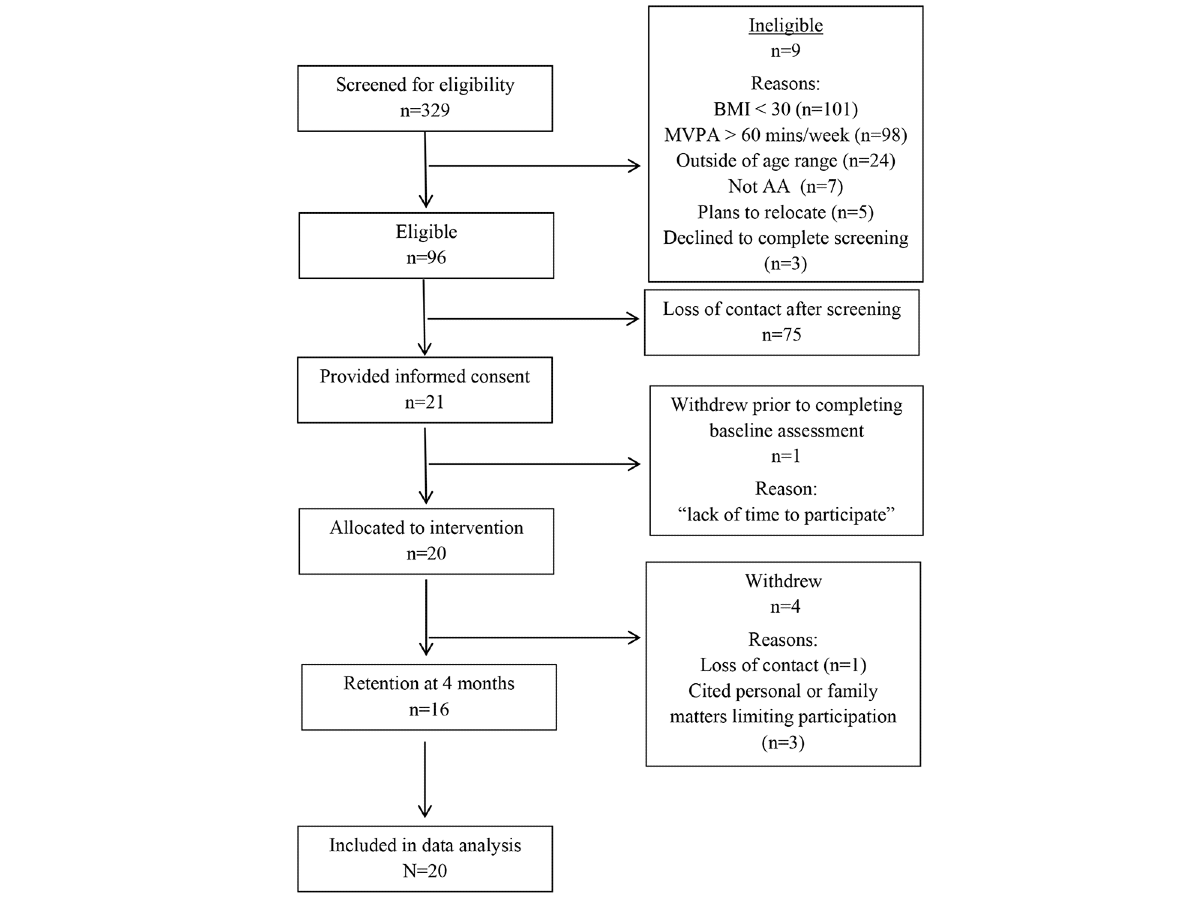
CONSORT (Consolidated Standards of Reporting Trials) diagram illustrating participant flow. AA: African American; MVPA: moderate- to vigorous-intensity physical activity.

Recruitment efforts resulted in 329 women being screened for eligibility. Of these, 96 women were eligible, and 21 provided informed consent to participate in the study. The low recruitment rate (22%) from eligibility to enrollment was due to individuals completing the web-based eligibility screening survey advertised on social media but not responding to follow-up inquiries from the research team for enrollment. One participant withdrew from the study before receiving the intervention, citing a lack of time to participate, resulting in 20 women receiving the intervention. In total, 16 participants provided follow-up data at 4 months (ie, 80% retention), with 1 participant’s completed follow-up data forms being lost by the project staff. The reasons for withdrawal at follow-up included loss of contact (n=2) and personal issues limiting study participation (n=2). Figure 1 provides detailed information regarding participant flow throughout the study, including reasons for ineligibility and withdrawal from the study.

Participants who began the intervention (n=20) had a mean age of 56.2 (SD 4.3) years and a mean BMI of 40.0 (SD 8.6) kg/m^2^. Overall, 45% (9/20) of women were married (n=8) or living in a marriage-like relationship (n=1), 30% (6/20) were either divorced (n=5) or separated (n=1), and 25% (5/20) were never married (n=5). In addition, 40% (8/20) of women attended some college or technical school, 35% (7/20) had a bachelor’s degree, and 25% (5/20) had a master’s degree. One participant reported an annual household income of <US $25,000, 30% (6/20) reported annual household incomes of US $25,000-US $50,000, 45% (9/20) reported incomes of US $50,001-US $100,000, and 20% (4/20) reported incomes >US $100,000.

### PA Outcomes

The PA outcomes are presented in Table 3. On the basis of Exercise Vital Sign Questionnaire, self-reported minutes per week of MVPA increased from a median of 20 minutes per week at baseline to 50 minutes per week at 4 months (*r*=0.503; *P*<.001). Weekly estimated energy expenditure assessed by the REGICOR indicated comparable increases for total weekly energy expenditure (baseline median=58.04 MET-min per week; 4-month median=265.91 MET-min per week; *r*=0.407; *P*=.008), with changes in light-intensity (*r*=0.423; *P*=.005) and moderate-intensity activities (*r*=0.512; *P*<.001) accounting the majority of this change.

**Table 3:** Self-reported physical activity outcomes.

Variable	Baseline			4 months			*P* value^a^		Effect size^b^
	Mean (SD)	Median (range)	Mean (SD)		Median (range)				
Exercise vital sign (min per week)	27.00 (26.57)	20 (0-90)	65.50 (61.43)		50.00 (0-200)	<.001		0.503^c^	
REGICOR light energy expenditure (MET^d^-min per week)	36.83 (27.64)	37.30 (0-111.89)	84.15 (71.86)		55.94 (0-268.07)	.005		0.423^e^	
REGICOR moderate energy expenditure (MET-min per week)	27.39 (60.18)	0 (0-268.07)	86.66 (84.17)		67.02 (0-267.07)	<.001		0.512^c^	
REGICOR vigorous energy expenditure (MET-min per week)	78.05 (140.91)	6.12 (0-512.82)	93.46 (135.64)		9.03 (0-480.42)	.50		0.114^f^	
REGICOR total energy expenditure (MET-min per week)	142.27 (178.20)	58.04 (0-585.08)	264.26 (199.62)		265.91 (41.96-732.17)	.008		0.407^e^	

^a^Wilcoxon signed-rank *P* value for baseline to 4-month change.

^b^Pearson *r* effect size estimate.

^c^Effect size: large (0.50-1).

^d^MET: metabolic equivalent.

^e^Effect size: medium (0.30-0.49).

^f^Effect size: small (0.10-0.29).

### SCT Outcomes

Pre-post intervention changes in SCT constructs targeted by the intervention are presented in Table 4. The results showed enhancements in self-regulation (*r*=0.397; *P*=.01) and behavioral capability (*r*=0.440; *P*=.004) for PA over the 4-month intervention period. An unexpected decrease in exercise self-efficacy for PA was also observed (*r*=−0.364; *P*=.02). No pre- and postintervention changes were observed for outcome expectations (*r*=−.029; *P*=.87), social support from family (*r*=0.103; *P*=.55), or social support from friends (*r*=0.083; *P*=.62).

**Table 4:** Social cognitive theory outcomes.

Variable	Range^a^	Baseline			4 months			*P* value^b^		Effect size^c^
		Mean (SD)	Median	Mean (SD)		Median				
Outcome expectations	1-5	4.30 (0.89)	4.61	4.41 (0.49)		4.61	.87		−0.029	
Self-regulation	1-5	1.96 (0.46)	2.0	2.49 (0.89)		2.40	.01		0.397^d^	
Self-efficacy	1-5	3.95 (0.57)	3.86	3.64 (0.49)		3.63	.02		−0.364^d^	
Social support from friends	8-40	16.53 (9.92)	16.0	18.00 (9.64)		17.0	.62		0.083	
Social support from family	10-50	17.25 (7.91)	15.0	17.63 (7.47)		15.0	.55		0.103^e^	
Behavioral capability	1-5	2.30 (1.13)	2.50	3.40 (1.19)		4.0	.004		0.440^d^	

^a^Potential range for each survey measure.

^b^Wilcoxon signed-rank *P* value for changes from baseline to 4 months.

^c^Pearson *r* effect size estimate.

^d^Effect size: medium (0.30-0.49).

^e^Effect size: small (0.10-0.29).

### Acceptability and Feasibility

#### Fitbit Wear and Activity Tracking Feature

**Figure 2:**
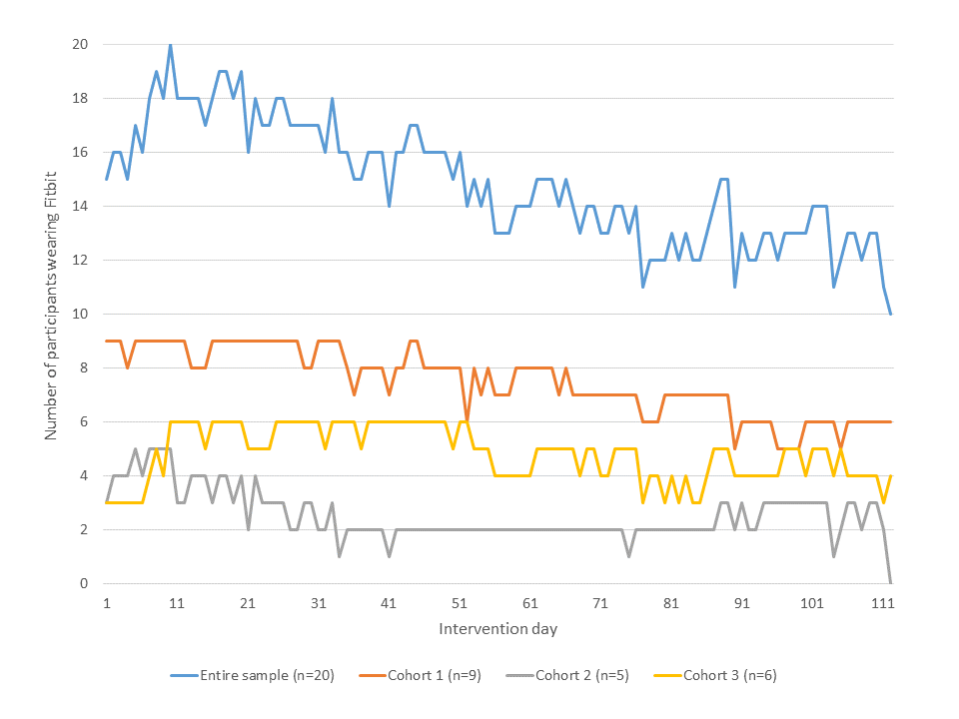
Fitbit wear by day and study cohort.

The median number of days participants wore the Fitbit device was 101.2 of the 112-day intervention period (ie, 91% of intervention days). Half of the sample (n=10) wore the device on more than 90% of intervention days, 3 participants wore the Fitbit between 75% and 90% of intervention days, 2 participants wore the device between 50% and 75% of intervention days, and 25% (5/20) of participants wore it for less than 50% of the intervention days. Daily Fitbit wear over time is presented in Figure 2, which shows a trend for decreasing Fitbit wear as the intervention period progressed.

Self-reported feedback regarding the activity tracking feature and the Fitbit activity monitor was generally favorable. Overall, 87% (13/15) of the sample indicated that the combined use of the Fitbit and activity tracking feature available on the app was *very motivating* (n=2), *motivating* (n=7), or *somewhat motivating* (n=4) for increasing PA; 2 participants reported that these features were *not motivating*. The majority of participants also indicated that the activity monitor was *very comfortable* (n=8) or *somewhat comfortable* (n=5) to wear; 1 participant reported that it was *a little comfortable*, and 1 indicated that it was *not comfortable*. With regard to frequency of accessing the activity tracking feature, 47% (7/15) of participants reported viewing the activity tracking feature more than 7 times per week, 7% (1/15) participant reported viewing the tracker 4-6 times per week, 27% (4/15) participants indicated viewing this feature 2-3 times per week, and 20% (3/15) participants reported viewing the feature no more than 1 time per week.

#### Multimedia PA Promotion Modules

**Figure 3:**
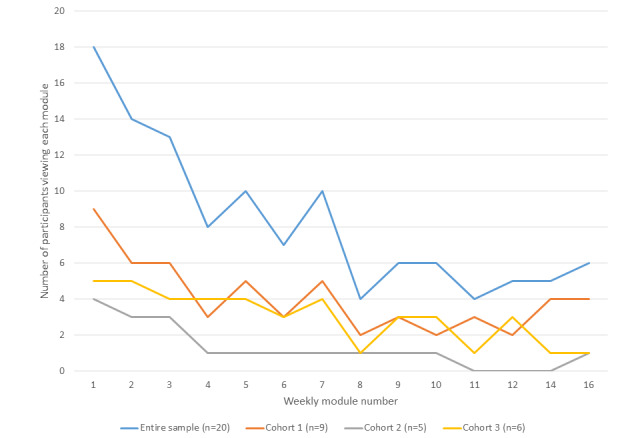
Module views by week and study cohort.

Among the 20 participants enrolled in the study, the median number of weekly multimedia intervention modules viewed was 4 (out of 14 possible). However, among participants who completed the intervention (n=16), the median number of modules viewed was 7. As illustrated in Figure 3, module viewing decreased as the 4-month study progressed. Videos embedded in these multimedia modules were viewed at a lower rate, with only 8 participants pressing the play video on these videos. Among these 8 participants, 3 viewed at least 75% (10/14) of the module videos, 1 viewed 50% (7/14) of the videos, and 4 viewed less than 50% (7/14) of the videos.

Participant feedback regarding multimedia modules indicated that 80% (12/15) of the participants found the modules to be *very helpful* (n=3), *helpful* (n=4), or *somewhat helpful* (n=5) for promoting PA. Three participants indicated that they were *not helpful* for promoting PA. In addition, 87% (13/15) of the participants indicated that they were *motivated* (n=4) or *somewhat motivated* (n=9) to be physically active as a result of weekly video and text modules; 2 participants said they were *not motivated*.

#### Discussion Board Features

**Figure 4:**
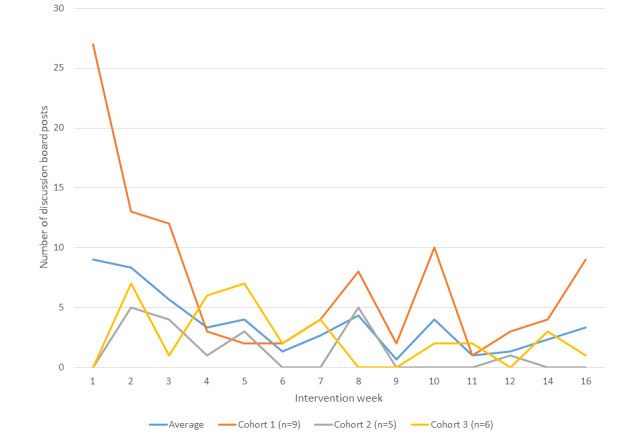
Discussion board posts by week and study cohort.

The number of discussion board posts varied by cohort and by week and is shown in Table 5 and graphically displayed in Figure 4. Similar to the trends observed for Fitbit wear and module viewing, discussion board posts decreased as time progressed. Participants reported accessing the message boards at varying frequencies: 20% (4/20) reported viewing the discussion boards 4-6 times per week, 20% (4/20) reported viewing 2-3 times per week, 15% (3/20) reported viewing 1 time per week, and 20% (4/20) reported viewing less than 1 time per week. Self-reported feedback from participants indicated that although the discussion boards were easy to use (12 of 15 participants indicated they did not have any difficulties using the discuss boards), their utility for encouraging PA was somewhat limited, as only 60% (9/15) of participants indicated the discussion boards were *helpful* (n=5) or *somewhat helpful* (n=4) for promoting PA; 40% (6/20) reported they were *not helpful*. Qualitative narratives indicated that participants felt that the discussion boards lacked interactivity and that additional activities were needed to enhance the discussion board feature and the social support components of the intervention. Quotes provided on the satisfaction survey illustrating the lack of interactivity among participants on the discussion boards included:

low interaction on the discussion board.

lack of camaraderie [on the discussion boards] among participants.

lack of interaction [on the discussion boards] with the group.

Participant narratives describing how we can improve the discussion boards and provisions of social support provided by the intervention included:

[have a member of the study team] encourage reaching out, connecting and partaking in the discussions.

[have] a discussion led by the program.

require a check-in [from study staff] 2-3 times per week and setup group exercise activities.

[have] a monthly meeting or web chat for participants or perhaps weekly.

maybe have [a study team member organize] a meet-up or challenge.

**Table 5:** Discussion board posts by cohort.

Week	Cohort 1 (n=9)		Cohort 2 (n=5)		Cohort 3 (n=6)	
	Total number participant posts	Frequency of participants posting	Total number participant posts	Frequency of participants posting	Total number participant posts	Frequency of participants posting
1	27	8	0	0	0	0
2	13	5	5	4	7	4
3	12	4	4	2	1	1
4	3	2	1	1	6	4
5	2	2	3	2	7	5
6	2	2	0	0	2	2
7	4	3	0	0	4	4
8	8	3	5	2	0	0
9	2	2	0	0	0	0
10	10	2	0	0	2	2
11	1	1	0	0	2	2
12	3	2	1	1	0	0
13	N/A^a^	N/A	N/A	N/A	N/A	N/A
14	4	3	0	0	3	2
15	N/A	N/A	N/A	N/A	N/A	N/A
16	9	4	0	0	1	1

^a^N/A: not applicable.

#### Text Messages

Text message delivery software indicated that text messages were delivered with a high level of fidelity. These data showed that all study text messages were delivered to 19 of the 20 participants. One text message was not delivered to one study participant (ie, sent but received an *undeliverable* response from Twilio). The research team was unable to identify the reason for this message not being deliverable, as the issue self-resolved, and the participant received all other study text messages. Feedback regarding the text messages indicated that 80% (12/15) of participants thought the text messages were *very helpful* (n=2), *helpful* (n=2), or *somewhat helpful* (n=8) for promoting PA.

#### Additional Feedback From Participants Regarding Acceptability and Feasibility of the Intervention

Among participants completing the satisfaction survey (n=15), 13 (87%) reported gaining knowledge about PA or exercise from the smartphone-delivered intervention and 14 (93%) indicated that they would recommend the intervention a friend. The one participant indicating she would not recommend the study to a friend stated that the intervention, “didn’t allow for building fitness connections.” This feedback, along with participant suggestions on how we can improve the discussion board features and social support components of the intervention, indicated the need to enhance the intervention to further promote connectedness and *camaraderie* among participants. Specifically, qualitative narratives provided by participants on the satisfaction survey suggested the need for a member of the study team or a PA coach to actively engage with participants and facilitate group-based social support for PA. Despite meaningful feedback provided by participants on how to improve the intervention, many participants expressed appreciation and positive sentiments, supporting the feasibility of the approach. Quotes illustrating this included:

I gained a lot and will continue to walk more and more.

Thank you! I really needed this. I was in a slump.

I like that it [the intervention] was interactive.

I enjoyed the study. It helped me keep moving.

keep all things [referring to the multiple components of the intervention], as everyone is different and [you] never know what motivates a person.

## Discussion

### Principal Findings

This study evaluated the acceptability and feasibility of a smartphone-delivered PA intervention among African American women. The results indicated that the smartphone-delivered approach increased self-reported PA and enhanced the theoretical mediators of intervention effects, including behavioral capability and self-regulation for PA. Objectively measured metrics of app use and intervention receipt, along with postintervention feedback from participants, highlighted several areas in which the intervention should be refined before larger-scale implementation of the intervention. The results add to the limited body of research on the use of mHealth PA interventions among midlife to older African American women.

The analyses showed a median increase of approximately 30 minutes of self-reported MVPA per week. Similar increases were also reported for the total weekly estimated energy expenditure. Effect size estimates for self-reported PA were comparable with those for self-reported PA outcomes reported in a previous eHealth and mHealth intervention of similar duration targeting African American women [41] as well as eHealth and mHealth PA interventions among obese women [42]. Evaluation of the SCT constructs targeted by the intervention showed enhancements in behavioral capability and self-regulation. We anticipate that these findings were related to the high level of module viewership during the early weeks of the intervention (ie, weeks 1-3; Figure 3), which largely included content focused on enhancing these psychosocial processes for the successful promotion of PA promotion (Table 1 shows the module content). These findings speak favorably for the intervention increasing knowledge and skill for PA and for participants enacting behavioral strategies to incorporate more PA into daily routine. An unexpected outcome was the decrease in self-efficacy. We speculate that participants may have been overly optimistic regarding their ability to increase PA at baseline, and once they attempted to increase PA, they realized that increasing their activity levels was not as easy as expected. Several participants alluded to this through comments provided on the satisfaction survey, including:

The program was good. It may have just been a bad time for me as it relates to the time commitment of working out.

I wish I had been as active as I inspired[sic] to be.

I work full-time and care for my mom after work, so time is precious.

In addition, although not specifically mentioned by participants in the satisfaction survey, we anticipate that the onset of the SARS-CoV-2 pandemic during the latter stages of intervention delivery for cohorts 2 and 3 also played a role in this outcome. The findings suggest the need to further explore how the intervention can be refined to leverage sources of self-efficacy for the successful promotion of PA.

The lack of changes from pre- to postintervention for outcome expectations may be a result of a ceiling effect, as participants reported relatively high scores on this measure (median scores of 4.61 out of 5, indicating favorable outcome expectations for PA) at both time points. Weak pre- versus postintervention changes in social support from family or friends were unsurprising, considering the lack of engagement in the app’s discussion board feature and qualitative feedback provided by participants. Together, these outcomes highlight a key area for intervention refinement. When originally developed, the purpose of the discussion board feature was to provide a venue for participants to interact with each other and to provide and receive social support. Weekly prompts provided on the discussion boards were included to initiate conversations among participants. However, our data show that these activities were not sufficient to promote social support for PA or engagement among participants on the *Smart Walk* app. Participants desired synchronous, more frequent, and interactive engagement with the study staff and other study participants. In particular, participants suggested that a member of the research team actively engage with them, provide frequent one-on-one check-ins for support and accountability, and organize group exercise sessions. Given that this is one of the first studies to examine an exclusively technology-mediated PA intervention among previously sedentary midlife to older African American with obesity, the findings provide important insights for researchers developing interventions for this population.

Objectively measured app use provided a great deal of insight regarding the feasibility and acceptability of the smartphone-delivered approach. The relatively high adherence to Fitbit wear, self-reported viewing of the activity tracking feature, and favorable outcomes regarding the motivation provided by these combined features suggest that it is feasible to provide midlife African American women with a Fitbit and allow them to track their activity. Nonetheless, we observed a decrease in Fitbit wear over the course of the study. In future trials, we will incorporate additional strategies to increase adherence to this study component, particularly among participants who demonstrate a decline in wear during the latter stages of intervention delivery. We were also able to deliver text messages to participants with a high degree of fidelity. However, a limitation of the study was that we did not include an item on the satisfaction survey assessing how many of the text messages participants read. Such information would have yielded greater insight into the receipt of the study text messages and will be incorporated into future studies.

Analytic tracking software showed that participants completing the intervention viewed half of the available weekly multimedia PA promotion modules, with a trend for decreased module viewing as the study progressed. Although decreased app use over time is a common issue reported in the mHealth literature [43], these outcomes may indicate the need to revise the modules to make them more interactive and to identify strategies to entice viewership (ie, reminders to view the modules for participants who have not viewed them). Identifying effective strategies to increase module viewing will increase the *dose* of the intervention received by participants, which, in theory, is expected to further enhance PA outcomes. However, this is not definitive. Additional work is needed to identify the optimal amount of intervention receipt needed to promote meaningful changes in PA.

### Limitations and Strengths

Limitations of the study include the small sample size and lack of a control group. Given the purpose of this study was to examine the feasibility of the intervention, rather than efficacy testing, the design was appropriate. Similarly, the multicomponent nature of the intervention does not allow us to draw conclusions as to whether one component is more effective than another for promoting PA. To answer this type of research question, a multiphase optimization strategy design may be warranted. Other limitations include the use of self-reported PA measures as study outcomes and that the latter stages of intervention delivery for cohorts 2 and 3 coincided with the early stages of the COVID-19 pandemic (February to April 2020). Given that this time was characterized by disruptions of daily routines, uncertainty regarding how the novel coronavirus was transmitted and the likelihood of being infected, and enhanced psychological stress [44,45], these issues likely impacted participants’ PA patterns, psychosocial outcomes (particularly self-efficacy for PA), app use, and attrition (3 of the 4 women lost to follow-up were enrolled in these cohorts). Likewise, safety concerns associated with virus transmission inhibited in-person data assessments at 4 months for participants in these 2 cohorts, limiting our ability to examine BMI and blood pressure outcomes among participants. Finally, we originally planned to include wrist-worn accelerometer-measured PA as an outcome measure. However, the collection of these data proved challenging among participants completing their 4-month follow-up assessments during the pandemic. Participants indicated limited interest in wearing the device due to COVID-19–related stress and ongoing disruptions of their daily activities. This resulted in less than half of the participants providing valid wear at the 4-month follow-up. Owing to the high rate of missing accelerometer data, objectively measured PA was not reported here.

Despite these limitations, this study has several strengths. This is one of the few studies to test a mHealth PA intervention among midlife to older African American women. These findings provide important insights into the use of mHealth technology to promote PA among this population at high risk for cardiometabolic diseases. Other strengths include the use of cultural tailoring and behavioral theory in the intervention design. Cultural tailoring is believed to enhance the salience and behavioral outcomes of a behavior change intervention [28,46,47], and numerous studies have shown that theoretically based behavior change interventions are more effective than those that are atheoretical [48-51]. These design considerations address the weaknesses noted in previous reviews of the PA promotion literature among African American women [41,52].

### Conclusions

The results provide preliminary support for the feasibility of the smartphone-based approach to increase PA among insufficiently active midlife to older African American women. However, before larger-scale implementation, several refinements to the intervention are necessary, including enhancing the social support components of the intervention. The findings will be used to inform future research using smartphone-based approaches to promote PA among midlife African American women.

## Abbreviations

MET: metabolic equivalent
mHealth: mobile health
MVPA: moderate- to vigorous-intensity physical activity
PA: physical activity
SCT: social cognitive theory

## References

[1] Hales CM, Carroll MD, Fryar CD, Ogden CL (2017). Prevalence of obesity among adults and youth: United States, 2015-2016. NCHS Data Brief.

[2] Benjamin EJ, Muntner P, Alonso A, Bittencourt MS, Callaway CW, Carson AP, Chamberlain AM, Chang AR, Cheng S, Das SR, Delling FN, Djousse L, Elkind MS, Ferguson JF, Fornage M, Jordan LC, Khan SS, Kissela BM, Knutson KL, Kwan TW, Lackland DT, Lewis TT, Lichtman JH, Longenecker CT, Loop MS, Lutsey PL, Martin SS, Matsushita K, Moran AE, Mussolino ME, O'Flaherty M, Pandey A, Perak AM, Rosamond WD, Roth GA, Sampson UK, Satou GM, Schroeder EB, Shah SH, Spartano NL, Stokes A, Tirschwell DL, Tsao CW, Turakhia MP, VanWagner LB, Wilkins JT, Wong SS, Virani SS, American Heart Association Council on EpidemiologyPrevention Statistics CommitteeStroke Statistics Subcommittee (2019). Heart Disease and Stroke Statistics-2019 Update: a report from the American Heart Association. Circulation.

[3] (2017). National Diabetes Statistics Report, 2017. Centers for Disease Control and Prevention.

[4] Curtin SC (2019). Trends in cancer and heart disease death rates among adults aged 45–64: United States, 1999–2017. National Vital Statistics Reports.

[5] (2008). 2008 physical activity guidelines for Americans.

[6] Williams WM, Yore MM, Whitt-Glover MC (2018). Estimating physical activity trends among blacks in the United States through examination of four national surveys. AIMS Public Health.

[7] Keller C, Larkey L, Distefano JK, Boehm-Smith E, Records K, Robillard A, Veres S, Al-Zadjali M, O'Brian A (2010). Perimenopausal obesity. J Womens Health (Larchmt).

[8] Rosano GM, Vitale C, Marazzi G, Volterrani M (2007). Menopause and cardiovascular disease: the evidence. Climacteric.

[9] Khoudary SR, Thurston RC (2018). Cardiovascular implications of the menopause transition: endogenous sex hormones and vasomotor symptoms. Obstet Gynecol Clin North Am.

[10] Buckingham SA, Williams AJ, Morrissey K, Price L, Harrison J (2019). Mobile health interventions to promote physical activity and reduce sedentary behaviour in the workplace: a systematic review. Digit Health.

[11] Buchholz SW, Wilbur J, Ingram D, Fogg L (2013). Physical activity text messaging interventions in adults: a systematic review. Worldviews Evid Based Nurs.

[12] Feter N, Santos TS, Caputo EL, da Silva MC (2019). What is the role of smartphones on physical activity promotion? A systematic review and meta-analysis. Int J Public Health.

[13] Joseph RP, Keller C, Adams MA, Ainsworth BE (2015). Print versus a culturally-relevant Facebook and text message delivered intervention to promote physical activity in African American women: a randomized pilot trial. BMC Womens Health.

[14] Kim BH, Glanz K (2013). Text messaging to motivate walking in older African Americans: a randomized controlled trial. Am J Prev Med.

[15] McCoy P, Leggett S, Bhuiyan A, Brown D, Frye P, Williams B (2017). Text messaging: an intervention to increase physical activity among african american participants in a faith-based, competitive weight loss program. Int J Environ Res Public Health.

[16] Zhang J, Jemmott Iii JB (2019). Mobile app-based small-group physical activity intervention for young african american women: a pilot randomized controlled trial. Prev Sci.

[17] (2017). Mobile fact sheet. Pew Research Center.

[18] Joseph RP, Ainsworth BE, Vega-López S, Adams MA, Hollingshead K, Hooker SP, Todd M, Gaesser GA, Keller C (2019). Rationale and design of Smart Walk: a randomized controlled pilot trial of a smartphone-delivered physical activity and cardiometabolic risk reduction intervention for African American women. Contemp Clin Trials.

[19] Smith A (2013). Smartphone ownership 2013. Pew Research Center.

[20] Coleman KJ, Ngor E, Reynolds K, Quinn VP, Koebnick C, Young DR, Sternfeld B, Sallis RE (2012). Initial validation of an exercise "vital sign" in electronic medical records. Med Sci Sports Exerc.

[21] Thomas S, Reading J, Shephard RJ (1992). Revision of the Physical Activity Readiness Questionnaire (PAR-Q). Can J Sport Sci.

[22] Joseph RP, Keller C, Vega-López S, Adams MA, English R, Hollingshead K, Hooker SP, Todd M, Gaesser GA, Ainsworth BE (2020). A culturally relevant smartphone-delivered physical activity intervention for African American women: development and initial usability tests of smart walk. JMIR Mhealth Uhealth.

[23] Tudor-Locke C, Han H, Aguiar EJ, Barreira TV, Schuna JM, Kang M, Rowe DA (2018). How fast is fast enough? Walking cadence (steps/min) as a practical estimate of intensity in adults: a narrative review. Br J Sports Med.

[24] Slaght J, Sénéchal M, Hrubeniuk TJ, Mayo A, Bouchard DR (2017). Walking cadence to exercise at moderate intensity for adults: a systematic review. J Sports Med (Hindawi Publ Corp).

[25] Reese JM, Joseph RP, Cherrington A, Allison J, Kim Y, Spear B, Childs G, Simpson T, Durant NH (2017). Development of participant-informed text messages to promote physical activity among African American women attending college: a qualitative mixed-methods inquiry. J Transcult Nurs.

[26] Joseph RP, Ainsworth BE, Mathis L, Hooker SP, Keller C (2017). Utility of social cognitive theory in intervention design for promoting physical activity among African-American women: a qualitative study. Am J Health Behav.

[27] Bandura A (1986). Social foundations of thought and action: a social cognitive framework.

[28] Resnicow K, Baranowski T, Ahluwalia JS, Braithwaite RL (1999). Cultural sensitivity in public health: defined and demystified. Ethn Dis.

[29] Molina L, Sarmiento M, Peñafiel J, Donaire D, Garcia-Aymerich J, Gomez M, Ble M, Ruiz S, Frances A, Schröder H, Marrugat J, Elosua R (2017). Validation of the regicor short physical activity questionnaire for the adult population. PLoS One.

[30] Joseph RP, Keller C, Adams MA, Ainsworth BE (2016). Validity of two brief physical activity questionnaires with accelerometers among African-American women. Prim Health Care Res Dev.

[31] Sallis JF, Pinski RB, Grossman RM, Patterson TL, Nader PR (1988). The development of self-efficacy scales for healthrelated diet and exercise behaviors. Health Educ Res.

[32] Sallis JF, Grossman RM, Pinski RB, Patterson TL, Nader PR (1987). The development of scales to measure social support for diet and exercise behaviors. Prev Med.

[33] Anderson ES, Winett RA, Wojcik JR, Williams DM (2010). Social cognitive mediators of change in a group randomized nutrition and physical activity intervention: social support, self-efficacy, outcome expectations and self-regulation in the guide-to-health trial. J Health Psychol.

[34] Anderson ES, Wojcik JR, Winett RA, Williams DM (2006). Social-cognitive determinants of physical activity: the influence of social support, self-efficacy, outcome expectations, and self-regulation among participants in a church-based health promotion study. Health Psychol.

[35] Resnick B, Zimmerman SI, Orwig D, Furstenberg AL, Magaziner J (2000). Outcome expectations for exercise scale: utility and psychometrics. J Gerontol B Psychol Sci Soc Sci.

[36] Vega WA, Sallis JF, Patterson T, Rupp J, Atkins C, Nader PR (1987). Assessing knowledge of cardiovascular health-related diet and exercise behaviors in Anglo- and Mexican-Americans. Prev Med.

[37] Kay MC, Carroll DD, Carlson SA, Fulton JE (2014). Awareness and knowledge of the 2008 Physical Activity Guidelines for Americans. J Phys Act Health.

[38] Hartman SJ, Nelson SH, Weiner LS (2018). Patterns of Fitbit use and activity levels throughout a physical activity intervention: exploratory analysis from a randomized controlled trial. JMIR Mhealth Uhealth.

[39] Joseph RP, Pekmezi D, Dutton GR, Cherrington AL, Kim Y, Allison JJ, Durant NH (2016). Results of a culturally adapted internet-enhanced physical activity pilot intervention for overweight and obese young adult African American women. J Transcult Nurs.

[40] Hsieh HF, Shannon SE (2005). Three approaches to qualitative content analysis. Qual Health Res.

[41] Joseph RP, Royse KE, Benitez TJ (2019). A systematic review of electronic and mobile health (e- and mHealth) physical activity interventions for African American and Hispanic women. J Phys Act Health.

[42] Cotie LM, Prince SA, Elliott CG, Ziss MC, McDonnell LA, Mullen KA, Hiremath S, Pipe AL, Reid RD, Reed JL (2018). The effectiveness of eHealth interventions on physical activity and measures of obesity among working-age women: a systematic review and meta-analysis. Obes Rev.

[43] Jee H (2017). Review of researches on smartphone applications for physical activity promotion in healthy adults. J Exerc Rehabil.

[44] Tull MT, Edmonds KA, Scamaldo KM, Richmond JR, Rose JP, Gratz KL (2020). Psychological outcomes associated with stay-at-home orders and the perceived impact of COVID-19 on daily life. Psychiatry Res.

[45] Park CL, Russell BS, Fendrich M, Finkelstein-Fox L, Hutchison M, Becker J (2020). Americans' COVID-19 stress, coping, and adherence to CDC guidelines. J Gen Intern Med.

[46] Resnicow K, Soler R, Braithwaite RL, Ahluwalia JS, Butler J (2000). Cultural sensitivity in substance use prevention. J Community Psychol.

[47] Resnicow K, Braithwaite R, Dilorio CK, Glanz K, Lewis F, Rimer B (2002). Applying theory to culturally diverse and unique populations. Health Behavior and Health Education: Theory, Research and Practice.

[48] Glanz K, Bishop DB (2010). The role of behavioral science theory in development and implementation of public health interventions. Annu Rev Public Health.

[49] Levy RL, Finch EA, Crowell MD, Talley NJ, Jeffery RW (2007). Behavioral intervention for the treatment of obesity: strategies and effectiveness data. Am J Gastroenterol.

[50] Davies CA, Spence JC, Vandelanotte C, Caperchione CM, Mummery WK (2012). Meta-analysis of internet-delivered interventions to increase physical activity levels. Int J Behav Nutr Phys Act.

[51] Noar SM, Benac CN, Harris MS (2007). Does tailoring matter? Meta-analytic review of tailored print health behavior change interventions. Psychol Bull.

[52] Whitt-Glover MC, Keith NR, Ceaser TG, Virgil K, Ledford L, Hasson RE (2014). A systematic review of physical activity interventions among African American adults: evidence from 2009 to 2013. Obes Rev.

